# Supplementary material for: Highly Efficient Transfer Hydrogenation of Biomass-Derived Furfural to Furfuryl Alcohol over Mesoporous Zr-Containing Hybrids with 5-Sulfosalicylic Acid as a Ligand
Source: Int J Environ Res Public Health. 2022 Jul 28;19(15):9221. doi: 10.3390/ijerph19159221 (PMC9368220; doi:10.3390/ijerph19159221)
Supplement: Supplementary file 1 [file ijerph-19-09221-s001.zip › ijerph-1793818-supplementary.pdf]

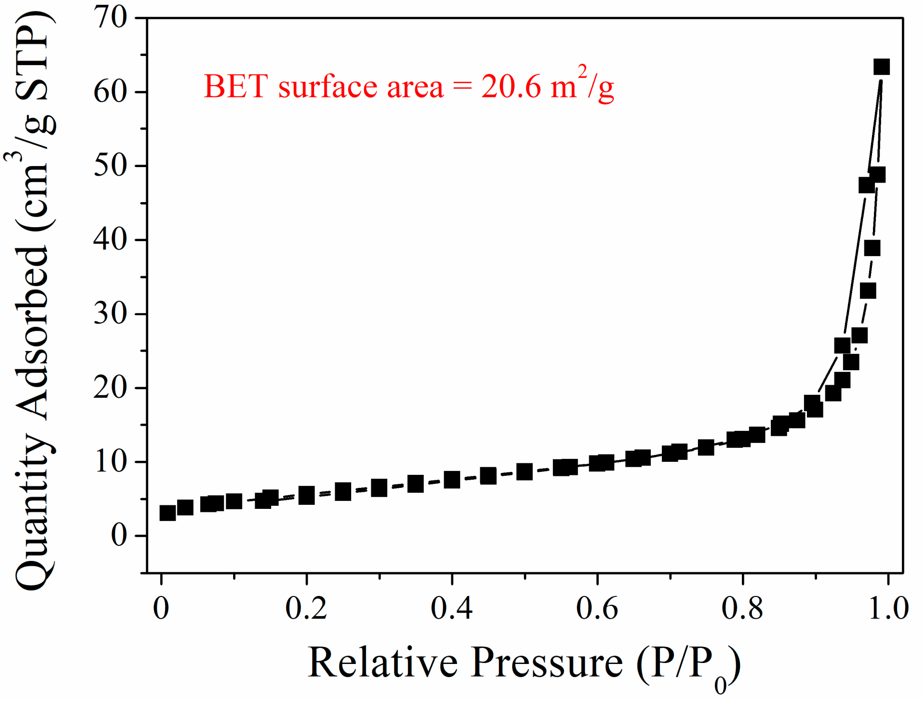

Figure S1. Nitrogen adsorption-desorption isotherm of nano-ZrO_2.

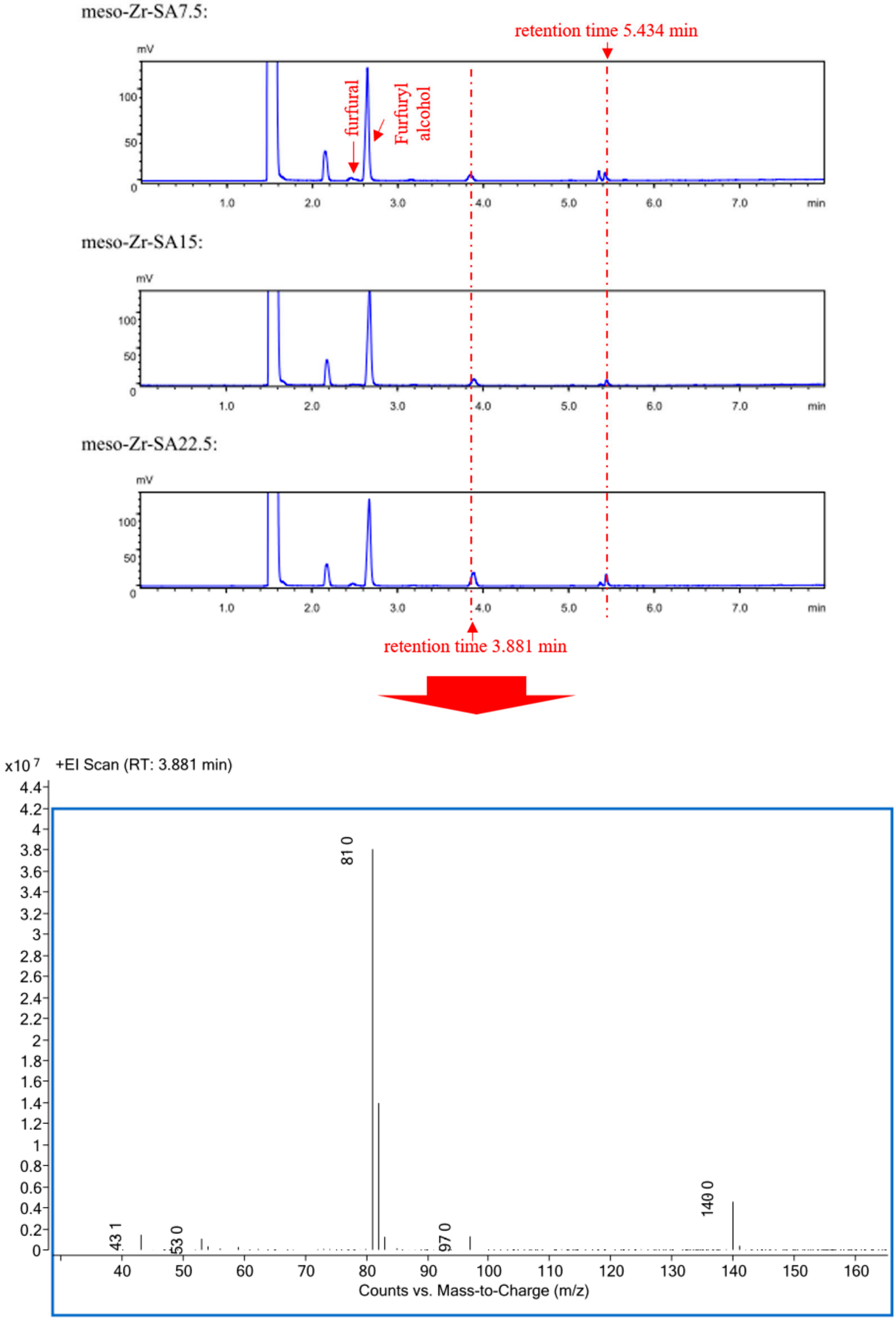

Figure S2. Gas chromatograms of samples obtained after reaction for 4 h over mesoporous Zr-hybrids along with the mass spectra of byproduct at retention time of 3.881 min. Reaction conditions: 1 mmol furfural, 100 mg catalyst, 110 °C.

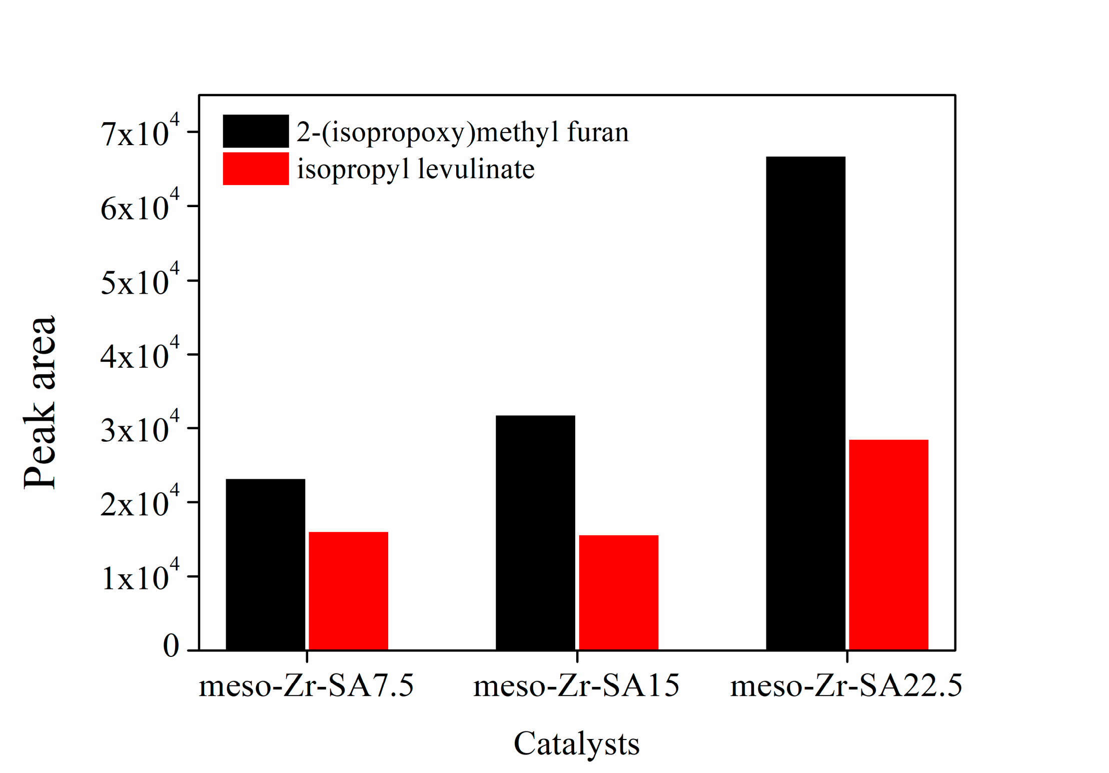

Figure S3. The peak area of each byproduct after reaction for 4 h over mesoporous Zr-hybrids.

Table S1. The contents of acid sites in nano-ZrO_2 basing on pyridine-adsorbed FTIR spectra at desorption temperature of 150 °C.

**Sample**	**BA[a] content (μmol/g)**	**LA[b] content (μmol/g)**	**BA+LA (μmol/g)**
nano-ZrO_2	7.0	71.3	78.3

[a] BA stands for Brønsted acid; [b] LA stands for Lewis acid

Table S2. MPV reduction of furfural to furfuryl alcohol over meso-Zr-SA15 at 130 and 150 °C

**Temperature (°C)**	**Time (h)**	**Furfural conversion (%)**	**Furfuryl alcohol yield (%)**	**Furfuryl alcohol selectivity (%)**
130	1	91.4	85.2	93.2
130	2	97.9	93.3	95.3
130	3	99.3	92.6	93.2
150	1	98.5	92.5	93.9
150	2	99.8	76.4	76.6

Table S3. Reusability of meso-Zr-SA15 in MPV reduction of furfural to furfuryl alcohol performed at reaction time of 2 h.

**Run**	**Furfural conversion (%)**	**Furfuryl alcohol yield (%)**	**Furfuryl alcohol selectivity (%)**
1	92.1	88.2	95.8
2	92.8	88.3	95.1
3	91.9	87.6	95.3
4	91.7	87.5	95.4
5	91.1	86.9	95.3
